# Brilliant angle-independent structural colours preserved in weevil scales from the Swiss Pleistocene

**DOI:** 10.1098/rsbl.2020.0063

**Published:** 2020-04-15

**Authors:** Luke T. McDonald, Suresh Narayanan, Alec Sandy, Vinodkumar Saranathan, Maria E. McNamara

**Affiliations:** 1 School of Biological, Earth and Environmental Sciences, University College Cork, Cork T23 TK30, Ireland; 2 Environmental Research Institute, University College Cork, Cork T23 XE10, Ireland; 3 Advanced Photon Source, Argonne National Laboratory, Argonne, IL 60439, USA; 4 Division of Science, Yale-NUS College, 138609, Singapore; 5 Department of Biological Sciences, National University of Singapore 117543, Singapore; 6 NUS Nanoscience and Nanotechnology Initiative (NUSNNI-NanoCore), National University of Singapore, 117581, Singapore; 7 Lee Kong Chian Natural History Museum, National University of Singapore, 117377, Singapore

**Keywords:** photonic nanostructures, structural colours, fossil colours, Pleistocene, weevils

## Abstract

Extant weevils exhibit a remarkable colour palette that ranges from muted monochromatic tones to rainbow-like iridescence, with the most vibrant colours produced by three-dimensional photonic nanostructures housed within cuticular scales. Although the optical properties of these nanostructures are well understood, their evolutionary history is not fully resolved, in part due to a poor knowledge of their fossil record. Here, we report three-dimensional photonic nanostructures preserved in brightly coloured scales of two weevils, belonging to the genus *Phyllobius* or *Polydrusus*, from the Pleistocene (16–10 ka) of Switzerland. The scales display vibrant blue, green and yellow hues that resemble those of extant *Phyllobius/Polydrusus*. Scanning electron microscopy and small-angle X-ray scattering analyses reveal that the subfossil scales possess a single-diamond photonic crystal nanostructure. In extant *Phyllobius/Polydrusus*, the near-angle-independent blue and green hues function primarily in crypsis. The preservation of far-field, angle-independent structural colours in the Swiss subfossil weevils and their likely function in substrate matching confirm the importance of investigating fossil and subfossil photonic nanostructures to understand the evolutionary origins and diversification of colours and associated behaviours (e.g. crypsis) in insects.

## 1. Introduction

Colour is a critical component of inter- and intraspecific visual communication in extant animals, including signalling strategies such as aposematism, mating displays and crypsis [1–4]. Biological photonic nanostructures––integumentary structures that scatter incident light––are a key evolutionary innovation [4–12]; they produce the most vibrant, highly saturated colours known in biological systems and can manipulate the directionality [9,13] and polarization properties [14,15] of scattered light. Such nanostructures are distributed broadly in extant insects and vary in their complexity, ranging from relatively simple multilayer reflectors to more complex three-dimensional architectures that include amorphous networks and highly ordered crystals [10,16–23]. In insects, three-dimensional photonic nanostructures occur exclusively in scale-bearing taxa [10,16–23], principally Lepidoptera [16,24,25], weevils [26,27], longhorn beetles [28,29] and, occasionally, scarabs [30].

Despite extensive research into the development, function and optical properties of three-dimensional photonic nanostructures in extant insects, and notwithstanding insights from modern phylogenetic analyses [31], the evolution of such structures is poorly resolved [22,25,32,33]. Fossil insects have the potential to inform on this issue but reports of fossil photonic nanostructures are rare. Structurally coloured fossil insects (beetles and moths) are known from Miocene and Eocene deposits, but preserve only multilayer reflectors [34,35]. Fossil scales possessing periodic nanostructures are preserved in Jurassic lepidopterans [36]; these scales feature a fused lumen and their predicted colour originates from thin-film interference [36,37]. Fossilized three-dimensional photonic nanostructures are known only from a single weevil (*Hypera diversipunctata*) from the Pleistocene of Canada that possesses a single-diamond (*Fd*-3*m*) photonic crystal (PC) structure within its scales [38]. Here, we report the preservation of bright blue and green scales on subfossil specimens of either *Phyllobius* or *Polydrusus* (Curculionidae: Entiminae) from Lobsigensee (late Pleistocene, Switzerland). Using scanning electron microscopy (SEM) and small-angle X-ray scattering (SAXS), we show that the bright colours of these scales are produced by a single-diamond three-dimensional PC structure and have optical properties consistent with substrate matching. The fossil nanostructures, therefore, represent intermediate stages in models for the evolution of three-dimensional photonic nanostructures and confirm the importance of the Pleistocene fossil insect record as a key source of data on the evolution of three-dimensional photonic nanostructures.

## 2. Material and methods

Specimens L150D-L and L150D-N were recovered from site L150D (a littoral pit) at the locality of Lobsigensee [39], a small lake on the western Swiss plateau (47°01′55″ N and 7°17′57″ E [40]). Prior to this study, the specimens were extracted from the sediment via sieving and kerosene flotation [39]. The specimens were recovered from the upper 0.825 m of site L150D, corresponding to an age of *ca* 10 000–13 000 BP [39]. A precise taxonomic determination was not possible, as the specimens comprise only elytra. However, the specimens were identified as belonging to either the genus *Phyllobius* or *Polydrusus* (S. Elias 2019, personal communication), which are closely related [41].

Optical micrographs of the specimens were taken using a Leica S8APO stereomicroscope fitted with a Leica DFC260 digital camera. For SEM, small (1 × 1 mm^2^) cuticle samples were mounted on an aluminium stub with carbon tape and sputter coated with Au/Pd. Samples were analysed using a FEI Inspect F50FE-SEM at 5 kV.

For SAXS, individual scales were detached manually from the cuticle and positioned in a custom-made aluminium sample holder between two pieces of 0.03 mm thick Kapton tape. Pinhole SAXS (15 µm horizontal × 15 µm vertical) data in transmission geometry were collected at beamline 8-ID-I at the advanced photon source (APS), Argonne National Laboratory as described elsewhere [22,25]. For additional methods, see the electronic supplementary material.

## 3. Results

**Figure 1.**
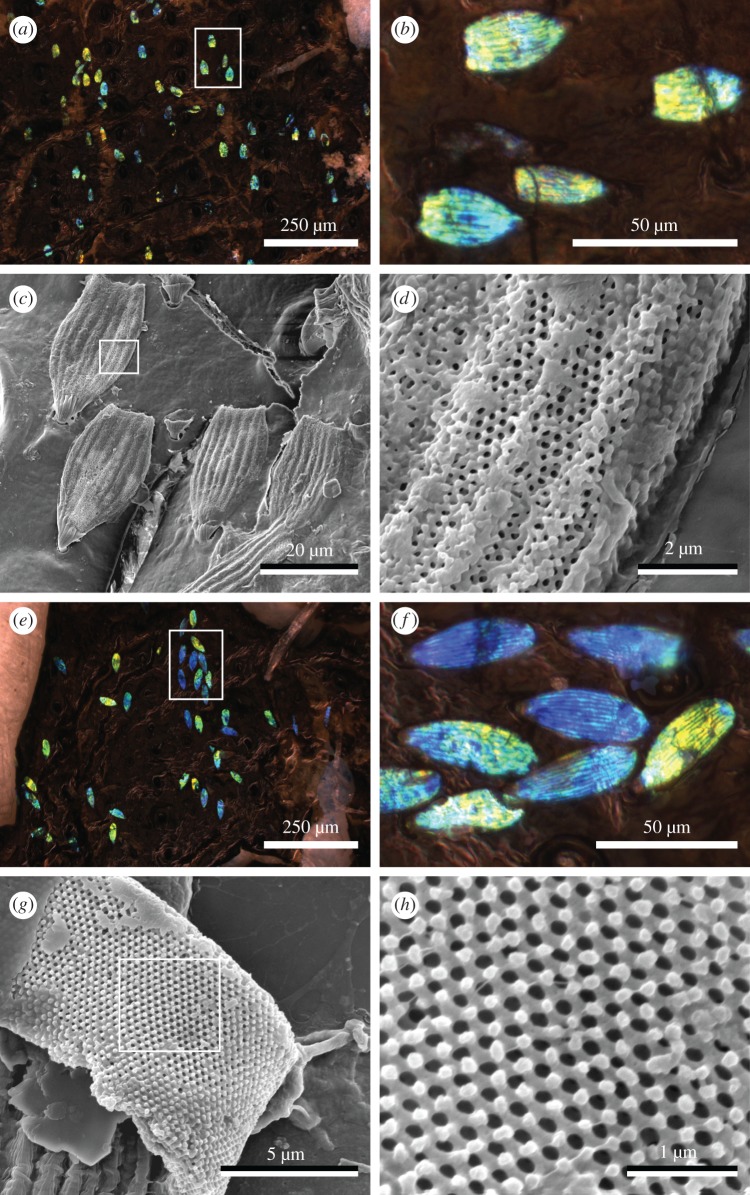
Pleistocene subfossil weevil scales from specimens L150D-L (*a*–*d*) and L150D-N (*e*–*h*) imaged using light microscopy (*a*,*b*,*e*,*f*) and SEM (*c*,*d*,*g*,*h*). Light micrographs show the preservation of scales preserving bright blue, green and yellow hues, while electron micrographs reveal three-dimensional photonic nanostructures. (*b*,*d*,*f*,*h*) Regions bounded by white boxes in (*a*,*c*,*e*,*g*; *a,e* rotated 90° clockwise), respectively.

Each subfossil specimen comprises a partial elytron of *Phyllobius/Polydrusus* exhibiting sparse bright blue to yellow-green scales that each show 8–10 parallel ridges oriented along the scale axis (figure 1*a,b*,e,f). Similar colours and axial ridges are evident in the scales of extant *Phyllobius* (see electronic supplementary material, figure S1).

SEM images (figure 1*c*,*d*,*g*,*h*) reveal that the scales of both subfossil specimens have an elongate spatulate shape (L150D-L: 30.81 ± 1.44 µm × 16.38 ± 1.83 µm; L150D-N: 44.62 ± 3.49 µm × 17.97 ± 1.53 µm) with rounded apical margins. Some scale margins, notably for specimen L150D-L, show the evidence of physical damage, while many scale pedicles are severed (see electronic supplementary material, figure S2). The paucity of preserved scales (relative to extant *Phyllobius*) thus likely reflects fracturing and loss of scales.

**Figure 2.**
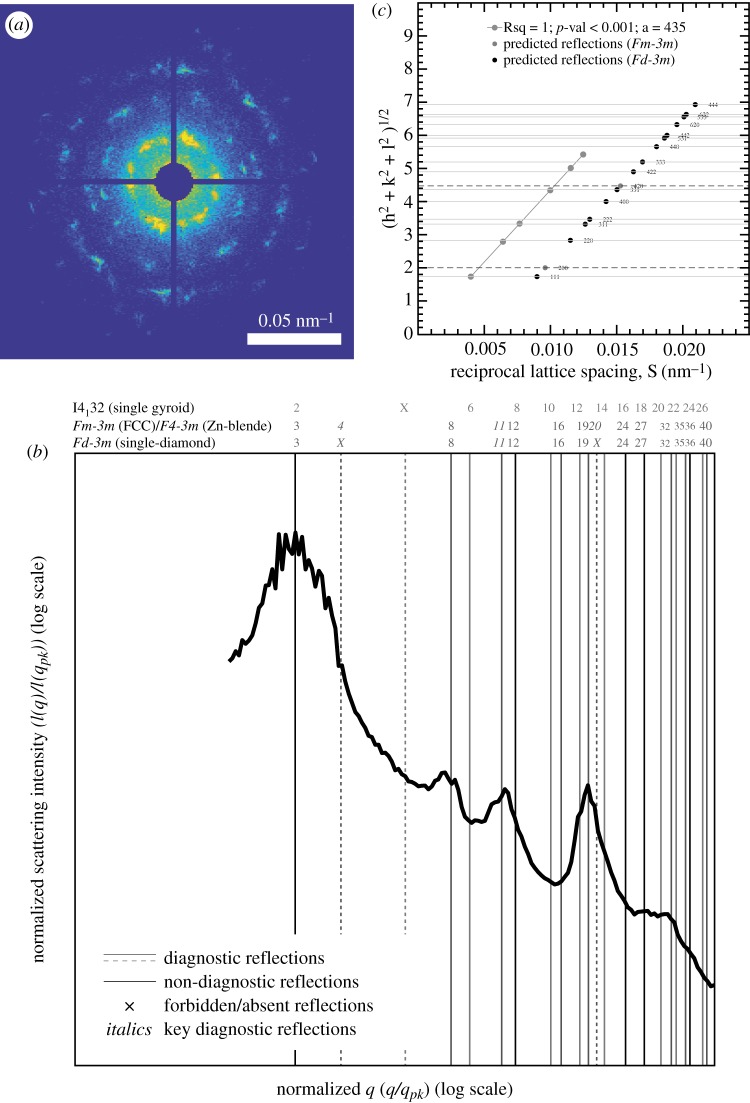
SAXS structural diagnosis of the three-dimensional photonic nanostructure in scales of specimen L150D-L. (*a*) Two-dimensional SAXS scattering pattern. (*b*) Normalized, azimuthally averaged SAXS profile integrated from the two-dimensional SAXS pattern in (*a*). Vertical lines denote expected Bragg peak positional ratios for various alternative cubic crystallographic space groups. Numbers above the lines are the squares of the moduli of the Miller indices (*hkl*) for the corresponding allowed reflections. The normalized positional ratios of the scattering peaks are indexed to the predictions of specific crystallographic space groups following IUCr conventions [35]. (*c*) Reciprocal lattice spacing *S* plotted against the moduli of the Miller indices confirms the cubic aspect of the nanostructure. The absence of experimentally measured Bragg scattering peaks coinciding with characteristic reflections from the *I*4_1_32 or *Fm*-3*m* symmetries is consistent with the single-diamond diagnosis for the subfossil nanostructure.

Both subfossil specimens preserve an ordered nanostructure within the scale lumen (figure 1*d*,*g*,*h*; see electronic supplementary material, figure S2). This nanostructure was characterized for specimen L150D-L using SAXS (figure 2). The two-dimensional scattering pattern (figure 2*a*) confirms the polycrystalline nature of the nanostructure, displaying a concentric series of discrete Bragg scattering peaks from variously oriented crystallite domains. The SAXS scattering profile (figure 2*b*) features discrete Bragg peaks with scattering wave vector positional ratios (*q/q_pk_*): √3, √8, √11 and √19 (where *q_pk_* = 0.025 nm^−1^ with a width (FWHM) of 0.0041 nm^−1^; *N* = 1). These peaks correspond to reflections from the (111), (220), (311) and (331) planes, which are consistent with the single-diamond (*Fd-*3*m*) space group symmetry [42]. Plotting the reciprocal lattice spacing (*S*) against the moduli of the assigned Miller indices (figure 2*c*) confirms the cubic aspect of the nanostructure and highlights the absence of scattering peaks at wave vector positional ratios √4 and √20 that distinguish the subfossil nanostructure from one with a face-centred cubic (*Fm*-3*m*) space group symmetry. In addition, it estimates the lattice constant for specimen L150D-L to be 435 nm. This result is consistent with SAXS structural measurements from extant *Phyllobius* and *Polydrusus* (433.41 nm and 440.02 nm, respectively; see electronic supplementary material in [22]).

Spectral measurements of the subfossil specimens exhibit reflectance peaks at 549 ± 5 nm (L150D-L) and 515 ± 4 nm (L150D-N), consistent with extant *Phyllobius* (520–580 nm; see electronic supplementary material, figure S3). We performed finite-difference time-domain (FDTD) simulations optimized to match the mean measured reflectance peak for specimen L150D-L, which indicate that its nanostructure has a mean refractive index (*n_avg_*) of 1.11, corresponding to a chitin (*n* = 1.56) volume fraction of 0.20 (cf. extant *Phyllobius *sp.** and *Polydrusus *sp.**, electronic supplementary material of [22]). SAXS reflectance predictions and photonic bandgap analyses calculated using these values are consistent with the observed spectrum for specimen L150D-L (see electronic supplementary material, figure S4).

## 4. Discussion

The low density of cuticular scales on the Lobsigensee specimens is not representative of extant *Phyllobius/Polydrusus*; this, plus the abundant fractured scales and/or scale pedicles, strongly indicates that additional scales were originally present but disarticulated from the elytra during decay and/or transport. This process is likely a major control on the fidelity of the fossil record of three-dimensional photonic structures. Despite the resistance of such structures to microbial decay and maturation at elevated temperatures and pressures [43], transport-induced scale loss is probably a major taphonomic filter in the early stages of fossilization. Critical information loss, therefore, occurs prior to the delivery of specimens to the site of deposition. Confounding factors may include transport-related biases linked to scale location, geometry and chitin filling fraction. Further, the small size of cuticular scales (particularly when incomplete) renders observation via light microscopy difficult; scales are rarely observed as disarticulated remains in sediments. An improved understanding of the fossil record and of the evolutionary origins of three-dimensional photonic nanostructures in fossil insects, therefore, requires careful investigation of fossil remains in order to identify any rare, small scales that have survived transport.

The subfossil *Phyllobius/Polydrusus* confirm that fossil three-dimensional PCs can produce vivid structural colours with minimal iridescence. Such nanostructures usually produce less angle-dependent colours than one-dimensional thin-film or multilayer reflectors due to the three-dimensional nature of the Brillouin zones of the underlying PCs [44]. In addition, iridescence is modulated by introducing disorder into the structure [27]. Although the Lobsigensee specimens possess an ordered single-diamond structure, the additive mixing of colours from differently oriented crystallites within the scales supresses iridescence at the macroscopic scale, giving rise to far-field near-angle-independent coloration [26,27,44].

The only other known example of fossil three-dimensional PCs (in the weevil *Hypera* from the Pleistocene of Canada) also comprises a single-diamond PC structure [38]. In contrast with *Phyllobius/Polydrusus*, the preserved *Hypera* scales exhibit an unremarkable reddish-brown macroscopic hue due to the larger lattice constant (*ca* 500 nm (*Hypera*); 435 nm (specimen L150D-L)) of the single-diamond structure, pointillistic mixing of colours from small (*ca* 2 µm wide) lattice domains and a higher chitin volume fraction (0.44 (*Hypera*); 0.20 (specimen L150D-L)) [38,45].

The diagnosis of a single-diamond PC structure in the subfossil *Phyllobius/Polydrusus* specimens is consistent with published SAXS data for extant *Phyllobius* and *Polydrusus* [22]. Single-diamond PCs are known only in weevils and are considered the dominant photonic structure in entimine weevils [22]. This is supported by recent phylogenetic studies [41,46], with the majority of weevils identified as possessing single-diamond PCs in [22] positioned in the Palaearctic-Oriental (including Phyllobiini) and Neotropical entimine clades. The scales of specimens lacking single-diamond structures instead contain amorphous structures or single gyroid PCs. The broader phylogenetic distribution of three-dimensional PCs in weevils points to a single origin at the base of a clade that groups Entiminae with the subfamily Cyclominae and the tribes Hyperini and Viticiini [33].

Entiminae are the most speciose weevil subfamily. Adults eat young shoots and leaves and typically deposit their eggs on plants, surface litter and/or soil. As a result, entimine weevils have evolved various strategies to facilitate crypsis, including substrate matching. Most (60%; *n* = 60) entimine weevils studied in [22] that possess single-diamond PCs have scales with green hues (blue-green to yellow-green). Given the near-angle-independent nature of the hues produced, three-dimensional PCs tuned to scatter green light provide efficient cryptic coloration in foliage over a broad range of angles. The fossil insect and pollen assemblages from Lobsigensee collectively indicate a temperate climate [40] and a eutrophic lake environment colonized by reeds, rushes and sedges [40]. It is therefore likely that the far-field angle-independent bright green hues of the *Phyllobius/Polydrusus* specimens performed a similar function to the matte brown of *H. diversipunctata* by facilitating cryptic substrate matching, in this case with vegetation.

Unlike other camouflage mechanisms used by insects (i.e. disruptive coloration and masquerade), crypsis minimizes the signal/noise ratio between body and background [3,47]. Disruptive coloration is often employed in habitats rich in bark, sand and soil, while crypsis and masquerade (as leaves/twigs) are often used in foliaceous environments [3]. In Coleoptera, the evolution of photonic nanostructures has been key to facilitating crypsis in foliaceous habitats as they lack the green-producing pigments, i.e. bilins, used in other insect orders (e.g. Lepidoptera, Mantodea, Orthoptera, Phasmida) [48]. Indeed, the diffuse far-field green reflectance of the *Entimus imperialis* weevil, which also results from a single-diamond PC, corresponds closely to the reflectance of green leaves [49]. Similarly, some lycaenid butterflies have green coloured ventral scales (containing a single gyroid three-dimensional PC nanostructure) that provide camouflage when perched with wings closed [50,51].

The key features of the *Phyllobius/Polydrusus* photonic nanostructure, i.e. a single-diamond PC tuned to scatter greenish hues, are a synapomorphy or shared-derived trait of a clade of broad-nosed weevils (predominantly Entiminae) as proposed by Seago *et al*. [31]. Such structures are considered evolutionarily derived relative to the amorphous or spongy nanostructures in brentids and higher weevils but primitive to structures that produce non-cryptic colours, such as in Pachyrrynchini and those with a single gyroid architecture [31]. This hypothesis, therefore, predicts that three-dimensional photonic nanostructures in the scales of higher weevil taxa (i.e. Curculionidae) will be dominated by amorphous nanostructures. In particular, the evolutionary origins of three-dimensional photonic nanostructures in weevil scales has been linked to the proliferation of angiosperms and the associated diversification of weevils in the Early Cretaceous [31,52,53]. Fossil weevils from the Early Cretaceous are thus prime targets for future studies on the evolution of three-dimensional PCs and are essential to test hypotheses on the origin and diversification of ancestral photonic nanostructures and their functions.

## Supplementary Material

Supplementary methods and figures

## Data accessibility

Data used for this analysis can be found either in this article, the electronic supplementary material, or openly accessed here: https://doi.org/10.5281/zenodo.3677700 (SAXS detector image, raw reflectance spectra).
