# Supplementary material for: Brilliant angle-independent structural colours preserved in weevil scales from the Swiss Pleistocene
Source: Biol Lett. 2020 Apr 15;16(4):20200063. doi: 10.1098/rsbl.2020.0063 (PMC7211455; doi:10.1098/rsbl.2020.0063)
Supplement: Supplementary methods and figures [file rsbl20200063supp1.pdf]

# Supplementary Information: Brilliant angle-independent structural colours preserved in weevil scales from the Swiss Pleistocene

Luke T. McDonald*, Suresh Narayanan, Alec Sandy, Vinodkumar Saranathan†, Maria E. McNamara

*luke.mcdonald@ucc.ie
†vinodkumar.saranathan@aya.yale.edu

## Extended materials and methods

### Microspectrophotometry:

Modern *Phyllobius* specimens (NMINH:1904.339.2 (*P. argentatus*), NMINH:1994.10.1 (*P. maculicornis*), NMINH:1994.10.2 (*P. roboretanus*) and NMINH:1968.38.1 (*P. virideaeris*)) from the National Museum of Ireland, Dublin were imaged using a Leica DM2700 M microscope (Universal LED illumination) coupled to a Leica MC190 HD digital camera. Reflectance spectra (both for fossil and modern specimens) were measured by positioning one end of a 600 μm optical fibre confocal to the image plane of the objective, with light guided to an Ocean Optics USB2000+ spectrometer. The peak intensity was averaged from measurements from 10 scales. A Labsphere Spectralon white reflectance standard served as reference for all reflectance measurements. In order to locate spectral reflectance peaks and extract the peak parameters and summary statistics, the microspectrophotometric data were loaded and analysed using the freely available Pavo 2.0 package (54) in the open-source environment, R (version 3.5.2).

### Finite-difference time-domain (FDTD) analysis:

We used the open-source FDTD software package MEEP (55) to simulate the optical reflectance in TE mode from the (111) plane of a single diamond network photonic nanostructure, with a fill fraction of 0.20 ($n_{avg}$ = 1.11) that was optimised to match the mean measured reflectance peak for specimen L150D-L (549 ± 5 nm). The (111) plane of the single diamond nanostructure was obtained from a level-set model of the single diamond (56), saved as a binaried, rescaled (to the values of permittivity ($\varepsilon$) for air and chitin: 1 and 2.4336) text file, and read directly into the computational cell using custom *Scheme* functions. We used a Gaussian line source to excite the lattice setup with periodic boundary conditions and perfectly matched layers (PML) in the direction of the plane wave propagation. We ran our simulations for an additional 50 time units until after the square amplitude at the transmittance plane had decayed to $1e^{-4}$ of its peak value, to ensure convergence. We also verified the MEEP software results by checking snapshots of wave propagation through the computational cell.

### Photonic bandgap calculation:

We used the MIT photonic bandgap (MPB) package (57) to calculate the first eight bands of a single diamond network photonic nanostructure in face-centred cubic basis, with a fill

fraction of 0.20 ($n_{avg}$ = 1.11), and using a mesh-size of 5 and a resolution of 32 to discretise the unit cell. The dielectric function was generated using the diamond level-set equation (56).

**SAXS reflectance prediction:**

The SAXS predicted reflectance follows from Bragg's law (25,58) and is given by mapping the X-ray scattering intensity from scattering wavevector ($q$) to wavelength space ($\lambda$), by choosing a value of 1.11 (informed by the FDTD optical simulations for specimen L150D-L) for the average refractive index ($n_{avg}$), corresponding to a chitin filling fraction of 0.20.

## Supplementary results

The experimentally measured reflectance spectrum of specimen L150D-L is shown (Figure S4a) alongside the reflectance spectra predicted using (i) SAXS structural analysis and (ii) a FDTD simulation of a single diamond network photonic nanostructure (informed by the SAXS derived lattice constant). The modelled spectra exhibit close agreement with the mean measured spectrum in terms of both position and spectral profile. The SAXS derived lattice constant also enabled the photonic bandgap diagram of the single diamond nanostructure to be calculated (Figure S4b). The position and bandwidth of the Γ-L (111) pseudogap (highlighted in green) is consistent with the experimentally measured mean reflectance of specimen L150D-L, and the SAXS and FDTD predictions (Figure S4a).

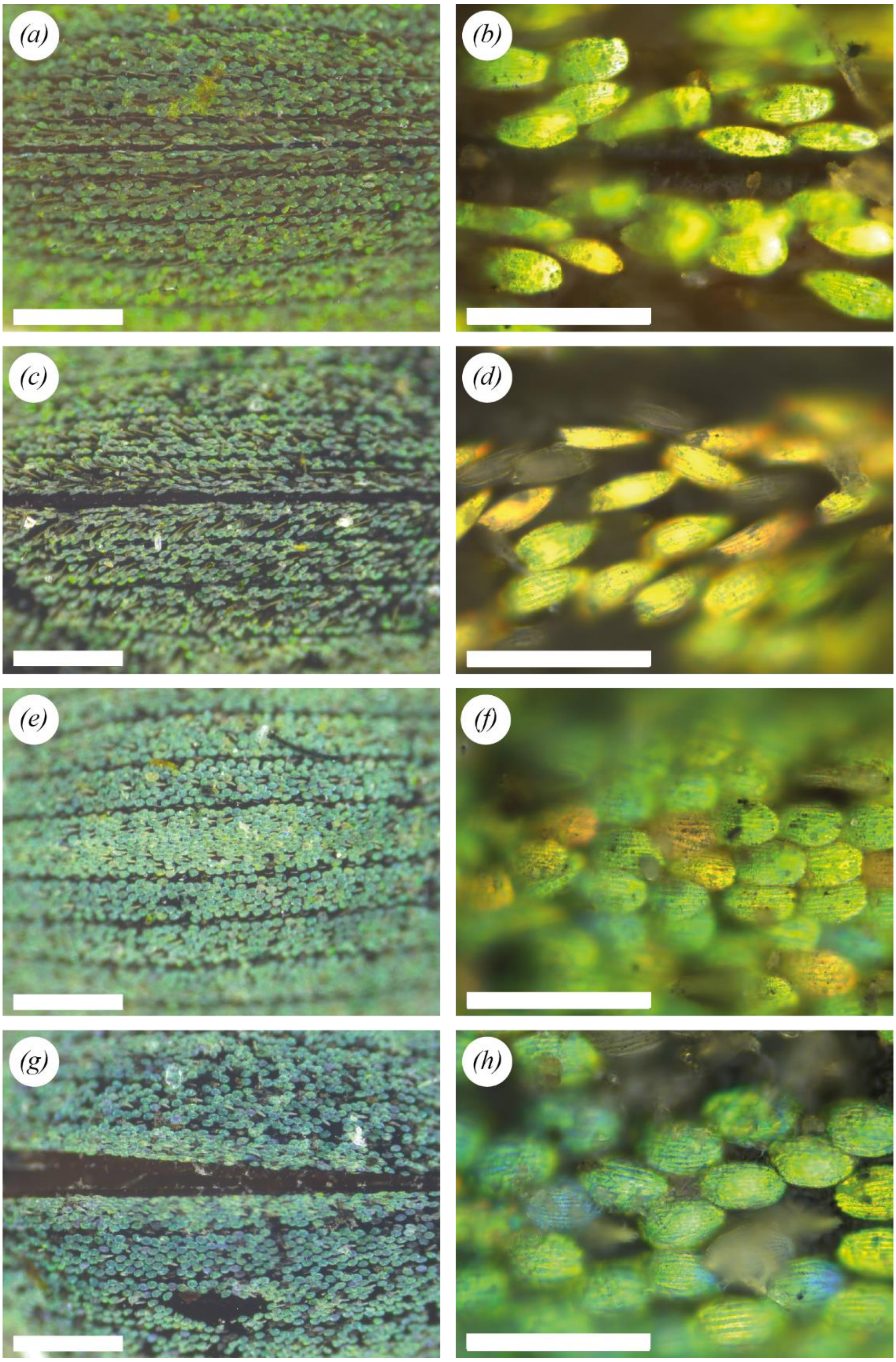


**Figure S1.** Optical micrographs showing scales from four extant species belonging to the genus *Phyllobius*: (*a*,*b*) *P. argentatus*; (*c*,*d*) *P. maculicornis*; (*e*,*f*) *P. roboretanus*; (*g*,*h*) *P. virideaeris*. Scale bars: (*a*,*c*,*e*,*g*) 200 μm; (*b*,*d*,*f*,*h*) 100 μm.

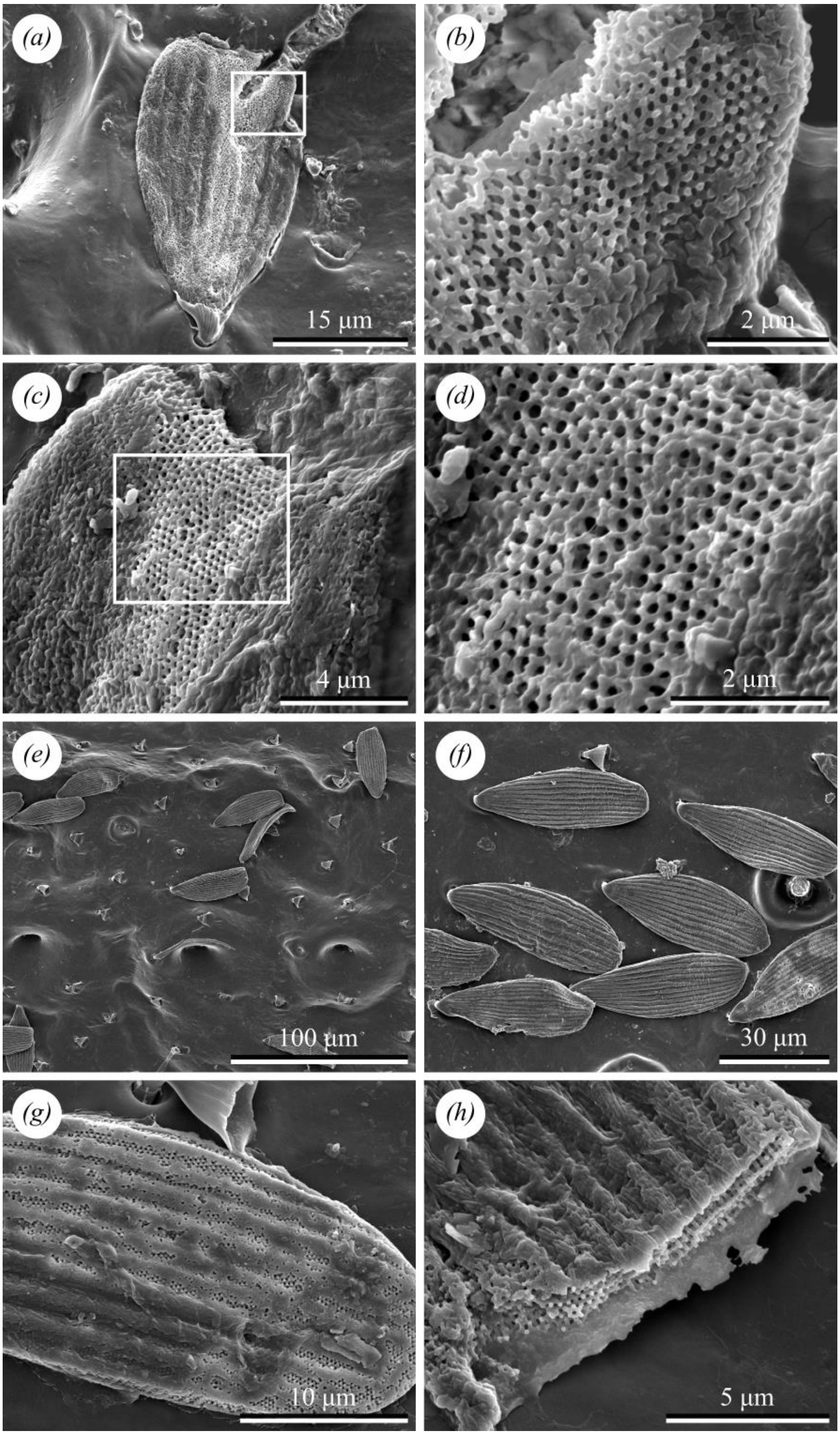


**Figure S2.** SEM images of (*a*–*d*) L150D-L and (*e*–*h*) L150D-N, showing features including damage to the apical margin of scales (*a*,*c*) and the exposed 3D photonic nanostructure (*b*–*d*) for specimen L150D-L, and numerous severed pedicles (*e*), well preserved scales (*f*, see figure 1*e*,*f*) and glimpses of the 3D photonic nanostructure for specimen L150D-N.

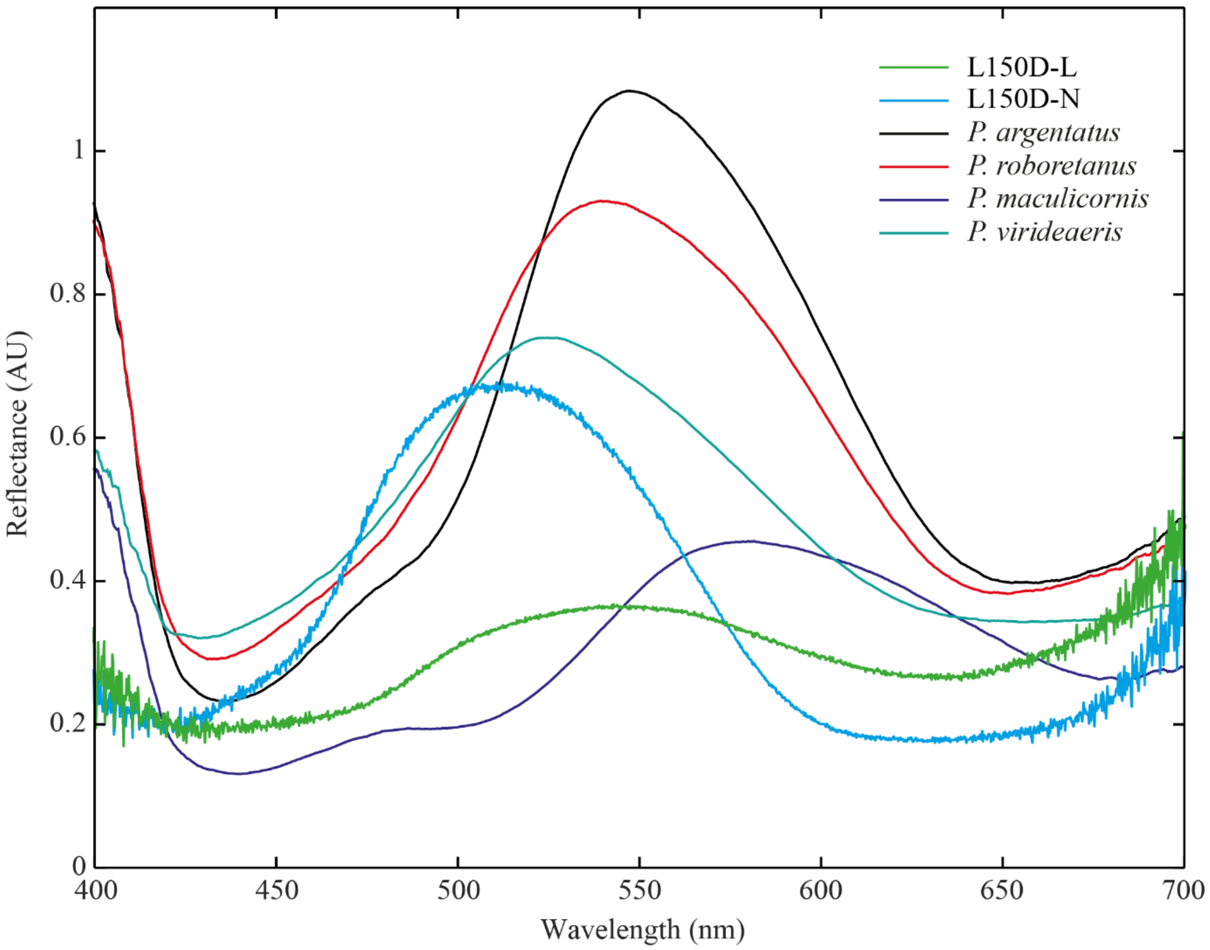


**Figure S3.** Experimentally measured reflectance spectra (beam spot diameter ca. 12 μm) comparing the optical response of subfossil *Phyllobius*/*Polydrusus* specimens L150D-L and L150D-N with extant *Phyllobius* species.

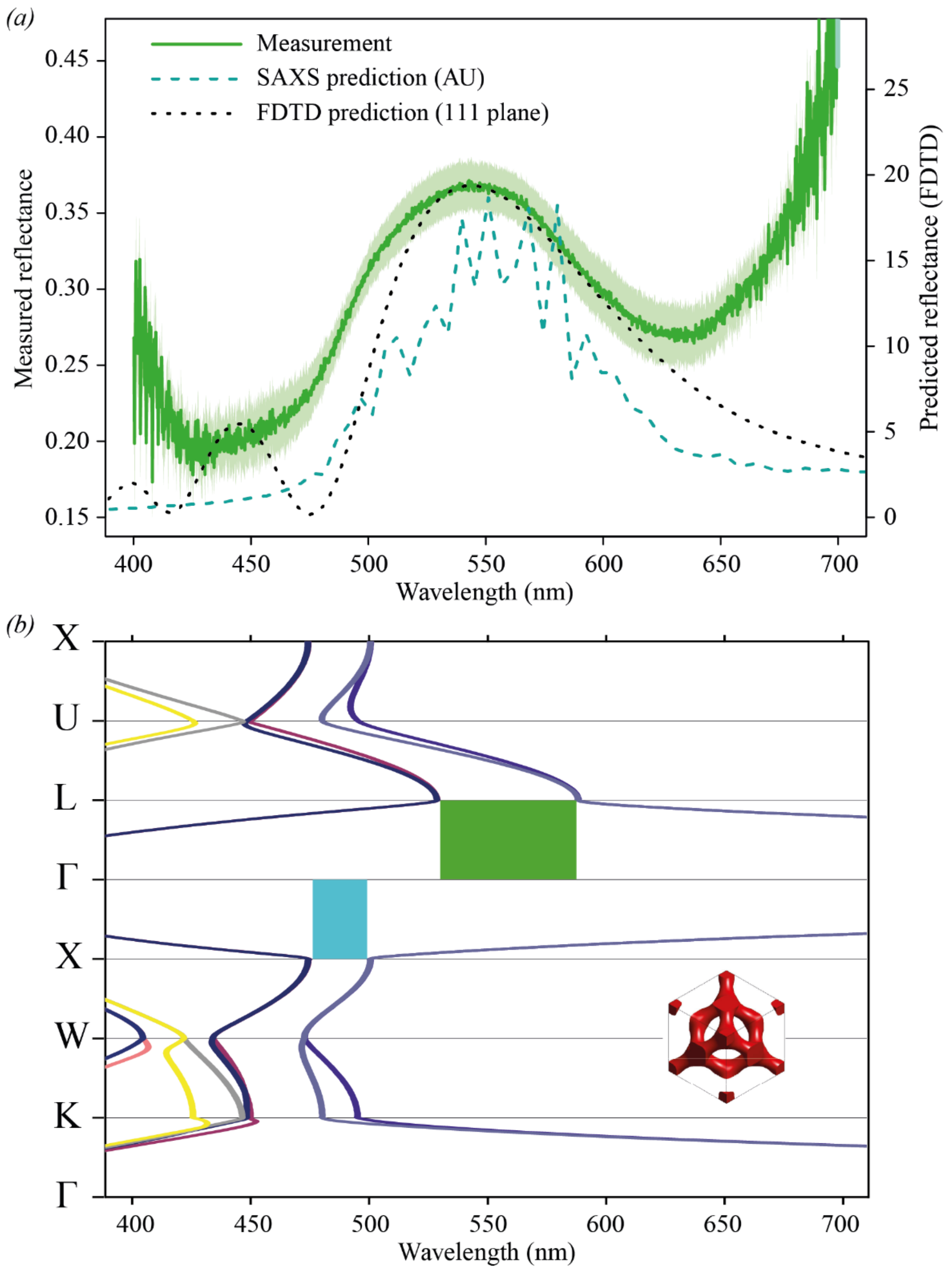


**Figure S4.** (*a*) A comparative plot showing the measured reflectance spectrum of specimen L150D-L alongside its reflectance as predicted by the SAXS analysis and the FDTD modelled reflectance of a single diamond nanostructure simulated using the SAXS derived lattice constant of specimen L150D-L ($a$ = 435 nm). (*b*) Photonic bandgap diagram of the simulated single diamond (*Fd*-3*m*) structure with a 20% chitin filling fraction shown in wavelength space ($\lambda = a/f$, where $a$ is the lattice parameter measured using SAXS and $f$ is the frequency). The presence of two relatively closely spaced gaps along the Γ-L (111) and Γ-X (100) directions are highlighted in green and blue, respectively. The width and midgap wavelength of the Γ-L (111) pseudogap are consistent with the reflectance measurement and predictions from SAXS and FDTD in (a). Inset: schematic of the single diamond (*Fd*-3*m*) crystal structure.
